# Supplementary material for: Meta-Analysis and Gene Set Enrichment Relative to ER Status Reveal Elevated Activity of MYC and E2F in the “Basal” Breast Cancer Subgroup
Source: PLoS One. 2009 Mar 9;4(3):e4710. doi: 10.1371/journal.pone.0004710 (PMC2650420; doi:10.1371/journal.pone.0004710)
Supplement: Dataset S1 — GSEA results from first screen. The file contains a zipped archive of the HTML results of the second GSEA screen. Reports for enriched or depleted datasets may be accessed from the “index.html” link within the my_analysis.GseaPreranked.1219975950625.rpt.zip ZIP archive. Enrichment plots are available for each of the 50 most enriched and depleted gene sets. (13.32 MB ZIP) [file pone.0004710.s001.zip › my_analysis.GseaPreranked.1219975950625.rpt.zip!//CHR12Q14.html]

Details for gene set CHR12Q14[GSEA]

|  || Dataset | zSco.ERneg.to.ERpos\_collapsed |
| Phenotype | NoPhenotypeAvailable |
| Upregulated in class | na\_neg |
| GeneSet | CHR12Q14 |
| Enrichment Score (ES) | -0.47115004 |
| Normalized Enrichment Score (NES) | -1.5898954 |
| Nominal p-value | 0.024324324 |
| FDR q-value | 0.13179567 |
| FWER p-Value | 0.999 |
Table: GSEA Results Summary

  

Fig 1: Enrichment plot: CHR12Q14      
 Profile of the Running ES Score & Positions of GeneSet Members on the Rank Ordered List

  

| PROBE | GENE SYMBOL | GENE\_TITLE | RANK IN GENE LIST | RANK METRIC SCORE | RUNNING ES | CORE ENRICHMENT || 1 | XPOT |  |  | 1517 | 2.930 | -0.0300 | No |
| 2 | WIF1 |  |  | 1544 | 2.907 | 0.0515 | No |
| 3 | GLIPR1 |  |  | 2439 | 2.229 | 0.0483 | No |
| 4 | AVPR1A |  |  | 4755 | 1.235 | -0.0903 | No |
| 5 | USP15 |  |  | 6734 | 0.577 | -0.2225 | No |
| 6 | TSPAN8 |  |  | 7315 | 0.378 | -0.2553 | No |
| 7 | TSFM |  |  | 7387 | 0.349 | -0.2506 | No |
| 8 | MMP19 |  |  | 7676 | 0.239 | -0.2654 | No |
| 9 | KIAA0984 |  |  | 7823 | 0.180 | -0.2712 | No |
| 10 | C12ORF31 |  |  | 7853 | 0.165 | -0.2687 | No |
| 11 | NUP107 |  |  | 8113 | 0.067 | -0.2862 | No |
| 12 | NAV3 |  |  | 8341 | -0.012 | -0.3030 | No |
| 13 | CCT2 |  |  | 8514 | -0.085 | -0.3135 | No |
| 14 | FAM119B |  |  | 8562 | -0.106 | -0.3139 | No |
| 15 | KCNC2 |  |  | 9043 | -0.329 | -0.3406 | No |
| 16 | OSBPL8 |  |  | 9451 | -0.539 | -0.3557 | No |
| 17 | LEMD3 |  |  | 9918 | -0.793 | -0.3680 | No |
| 18 | GNS |  |  | 10327 | -1.046 | -0.3686 | No |
| 19 | TMEM5 |  |  | 11691 | -2.210 | -0.4077 | Yes |
| 20 | CAND1 |  |  | 11831 | -2.352 | -0.3506 | Yes |
| 21 | PPM1H |  |  | 11915 | -2.452 | -0.2864 | Yes |
| 22 | TBK1 |  |  | 12180 | -2.817 | -0.2254 | Yes |
| 23 | MON2 |  |  | 13075 | -4.730 | -0.1568 | Yes |
| 24 | TMBIM4 |  |  | 13262 | -6.100 | 0.0044 | Yes |
Table: GSEA details [plain text format]

  

Fig 2: CHR12Q14: Random ES distribution      
 Gene set null distribution of ES for **CHR12Q14**
